# Are Circulating Mg^2+^ Levels Associated with Glucose Tolerance Profiles and Incident Type 2 Diabetes?

**DOI:** 10.3390/nu11102460

**Published:** 2019-10-14

**Authors:** Rosangela Spiga, Gaia Chiara Mannino, Elettra Mancuso, Carolina Averta, Claudia Paone, Mariangela Rubino, Angela Sciacqua, Elena Succurro, Francesco Perticone, Francesco Andreozzi, Giorgio Sesti

**Affiliations:** 1 Department of Medical and Surgical Sciences, University Magna Graecia of Catanzaro, 88100 Catanzaro, Italy; rosangela.spiga@gmail.com (R.S.); gaiamannino@gmail.com (G.C.M.); elettramancuso@gmail.com (E.M.); carolinaaverta90@gmail.com (C.A.); cla.31@libero.it (C.P.); marubino83@gmail.com (M.R.); sciacqua@unicz.it (A.S.); succurro@unicz.it (E.S.); perticone@unicz.it (F.P.); 2 Department of Clinical and Molecular Medicine, University Sapienza of Rome, 00189 Rome, Italy; giorgio.sesti@uniroma1.it

**Keywords:** magnesium, type 2 diabetes, glucose tolerance, survival analysis

## Abstract

Magnesium (Mg^2+^) is an enzyme co-factor that plays a key role in many biochemical reactions, as well as in glucose metabolism. Clinical evidences have demonstrated that depletion of serum Mg^2+^ increases exponentially with the duration of type 2 diabetes mellitus (T2DM). Diabetes is associated with low Mg^2+^, and hypomagnesemia is associated with insulin resistance, inflammation, and increased risk for cardiovascular disease. In subjects at high risk of inflammation and insulin resistance, supplementation of Mg^2+^ alone ameliorates both phenotypes, slowing the development and progression of hepatic steatosis. We analyze the relationship between serum Mg^2+^ levels and the onset of T2DM in a large cohort of well-characterized adult white individuals participating in the CATAMERI study, who were reexamined after a mean follow-up of 5.6 ± 0.9 years. In our analysis we acquired a significant negative correlation between Mg^2+^ levels, fasting glucose, and 2h-post load glucose in subjects who underwent an OGTT. Moreover, Mg^2+^ levels correlated negatively with fasting insulin levels, and positively with the lipid profile. As for the detrimental effect of lower circulating Mg^2+^ levels, our data revealed a significant reduction of T2DM risk of about 20% for each 1 mg/dL increase of circulating Mg^2+^. The present results are consistent with the theory that Mg^2+^ supplementation could ameliorate insulin sensitivity reducing the risk to develop T2DM.

## 1. Introduction

Mg^2+^ is one of the most abundant elements in nature, and its importance for human health has been recognized since the early 1900s [1]. In average, each person carries approximately 24 g (1 mol) of Mg^2+^, which is mainly stored in the bones and within the cells. Its homeostasis is only marginally affected by hormonal regulation, and Mg^2+^ levels in the blood strongly depend on the dynamic balance between intestinal absorption and renal excretion, whereas only about one third of the bone reservoir is exchangeable [2]. Nonetheless, the circulating concentration of Mg^2+^ is tightly controlled (range 0.9–1.0 mM), 70–80% of total Mg^2+^ circulates in the biologically active ionized (free) form, while the residual portion is bound to carrier proteins (e.g., albumin) (20–30%) or anions (e.g., phosphate, citrate, bicarbonate) (1–2%) [3,4]. Dietary intake of Mg^2+^ through food is generally considered sufficient to satisfy the recommended daily allowance of 420 mg for men and 320 mg for women [5].

Mg^2+^ is an enzyme co-factor in many biochemical reactions, regulating a number of physiological functions such as muscle contraction, neuromuscular conduction, glycemic control, myocardial electrical activity, and blood pressure [6,7,8]. Moreover, at a molecular level, Mg^2+^ is required by all enzymes involved in phosphorylation/dephosphorylation processes, which are usually pivotal for the transduction of intracellular signaling involving cell survival and metabolism [3,9]. Hypomagnesemia is clinically defined at serum Mg^2+^ concentration <0.5 mM, and may be due to primary deficiency [3], reduced Mg^2+^ intake and/or increased depletion [9]. Secondary Mg^2+^ deficiency may develop as a consequence of various pathologies (e.g., diabetes mellitus type II, alcoholism, HIV/AIDS, acute myocardial infarction) and treatments (e.g., hypermagnesuric diuretics, digitalis, cardiopulmonary bypass) [10]. 

Primary chronic hypomagnesemia has been linked to increased risk of preclinical and clinical events related to the metabolic syndrome, including: Cardiovascular disease and atherosclerosis, glucose intolerance, insulin resistance, diabetes mellitus, endothelial dysfunction, alterations of the lipid pattern, obesity, atheroma formation, platelet aggregation/abnormal thrombosis, inflammation, oxidative stress, and cardiovascular mortality [10,11,12,13,14,15,16]. 

More specifically, the antiatherogenic effects of Mg^2+^ appear to involve the modulation of lipid metabolism and turnover [17,18,19], and in the intestinal tract, Mg^2+^ can form insoluble soaps with fatty acids and therefore prevent absorption of dietary fat [20]. Obese adults and children are chronically deficient in Mg^2+^, which may help to explain the insulin resistance observed in these patients [21,22]; indeed, Mg^2+^ plays an important role in glucose metabolism regulating insulin secretion from pancreatic β-cells [23], transduction of the insulin signaling, and it modulates active glucose transporter into peripheral tissues [24]. In subjects at high risk of inflammation and insulin resistance, supplementation of Mg^2+^ alone ameliorates both phenotypes [25,26], slowing the development and progression of hepatic steatosis [27]. 

Consistent with these evidences, in 2005 a meta-analysis showed that the risk of T2DM was increased across all studies in subjects in the lowest Mg^2+^ consumption category [15], and that the risk was 8–13% lower per 100 mg/day increment in Mg^2+^ intake. To the best of our knowledge, only very few longitudinal epidemiological studies have analyzed the impact of serum Mg^2+^ levels on the risk of developing T2DM, and most of them appear inconsistent and inconclusive.

For this reason, in the present study, we point to analyze the relationship between serum Mg^2+^ levels and the onset of T2DM in a large cohort of well-characterized adult white individuals participating in the CATAnzaro MEtabolic RIsk factors study (CATAMERIS), who were reexamined after a mean follow-up of 5.6 ± 0.9 years.

## 2. Materials and Methods 

### 2.1. Study Population

The study group consisted of 589 white Caucasian subjects (288 men and 301 women), enrolled in the CATAMERIS, an observational study assessing cardio-metabolic risk in individuals carrying at least one risk factor including overweight/obesity, hypertension, dyslipidemia, dysglycemia, and family history for T2DM. The CATAMERI study is a metabolic disease prevention campaign dedicated to the identification and characterization of cardio-metabolic risk factors [28,29]. At the baseline, subjects, aged 21–75 years (mean 47 ± 13), were excluded if they had a history of cardiovascular disease, chronic gastrointestinal diseases, chronic pancreatitis, history of any malignant disease, history of alcohol or drug abuse, liver or kidney failure and treatments able to modulate glucose metabolism, including hypoglycemic agents. All subjects were subjected to anthropometrical evaluation before undergoing an oral glucose tolerance test (OGTT): Weight, height, body mass index (BMI), and waist circumference (WC) were measured, and body composition was evaluated by bioelectrical impedance. After a 12-h fast, a 75-g OGTT was performed with 0, 30, 60, and 120 min sampling for plasma glucose and insulin levels measurements. In accordance to the American Diabetes Association (ADA) criteria [30], subjects were classified into three groups according to the glucose tolerance status as having normal glucose tolerance (NGT) when fasting plasma glucose (FPG) was <7.0 mmol/L (100 mg/dL) and 2-h post-load <7.8 mmol/L (140 mg/dL), impaired fasting glucose (IFG) when FPG was 5.6–6.9 mmol/L (100–125 mg/dL) and 2-h post-load <7.8 mmol/L (140 mg/dL), impaired glucose tolerance (IGT) when FPG was <5.6 mmol/L (100 mg/dL) and 2-h post-load was 7.8–11.0 mmol/L (140–199 mg/dL) and diabetes when FPG was >7.0 mmol/L (126 mg/dL) and/or 2-h plasma glucose >11.1 mmol/L (200 mg/dL). 

For longitudinal study analysis, non-diabetic subjects who completed the baseline measurements were invited to a follow-up (FU) visit, or contacted by a telephonic survey, in order to evaluate their metabolic condition and to establish the time of the onset of T2DM. The study was approved by local Institutional Ethics Committees of University “Magna Graecia” of Catanzaro (approval code: 2012.63). Written informed consent was obtained from each subject in accordance with principles of the Declaration of Helsinki.

### 2.2. Calculation

Two indexes of insulin secretion were calculated from the OGTT data using the Stumvoll index (first-phase secretion = 1283 + 1.829 × Ins30 − 138.7 × Gluc30 + 3.772 × Ins0) [31], where Ins is insulin and Gluc is glucose, and early-phase insulin secretion was assessed by the ratio of insulin AUC0-30 to glucose AUC0-30 (InsAUC30/GluAUC30) during the OGTT [32]. The estimated glomerular filtration rate (e-GFR) was calculated by using the CKD-EPI equation [33]: eGFR = 141 xmin(Scr/k, 1)α xmax(Scr/k, 1) − 1.209 × 0.993Age × 1.018 (if female), where Scr is serum creatinine, k is 0.7 for females and 0.9 for males, α is −0.329 for females and −0.411 for males, min indicates the minimum of Scr/k or 1, and max indicates the maximum of (Scr/k or 1). Insulin sensitivity was evaluated using the Matsuda Index (insulin sensitivity index, ISI) calculated as follows: 10,000/square root of (fasting glucose (mmol/L) × fasting insulin (mU/L)) × (mean glucose × mean insulin during OGTT). The homeostasis model assessment (HOMA) index was calculated as fasting insulin × fasting glucose/22.5 [34].

### 2.3. Laboratory Determinations

HbA1c was measured with high performance liquid chromatography using a National Glycohemoglobin Standardization Program (NGSP) certified automated analyzer (Adams HA-8160 HbA1C analyzer, Menarini, Italy). Glucose, triglycerides, total, and high-density lipoprotein (HDL) cholesterol concentrations were determined by enzymatic methods (Roche, Basel, Switzerland). Plasma glucose was measured by the glucose oxidation method (Beckman Glucose Analyzer II; Beckman Instruments, Milan, Italy). Triglycerides and total and HDL cholesterol concentrations were measured by enzymatic methods (Roche Diagnostics, Mannheim, Germany). Plasma insulin concentration was determined by a chemiluminescence-based assay (Roche Diagnostics). Plasma insulin concentration was measured with a chemiluminescence-based assay (Immulite®, Siemens, Italy). Serum Mg^2+^ concentrations were measured by COBAS INTEGRA Magnesium, based on the colorimetric method assay (Roche Diagnostic, Mannheim, Germany).

### 2.4. Statistical Analysis

The results for continuous variables are given as means ± SD. Anthropometric and metabolic differences between groups were tested after adjusting for age, and gender using a general linear model with post hoc Bonferroni correction for multiple comparisons. The *X*^2^ test was used for categorical variables. Variables with skewed distribution (i.e., triglycerides, fasting insulin, HOMA index, and InsAUC30/GluAUC30) were log transformed to meet the normality assumption for statistical purposes. Correlation coefficients were calculated according to Pearson’s method. Partial correlation coefficients adjusted for age, sex, and BMI, were computed between variables. A multiple Cox regression analysis including age, sex, and BMI at the baseline as covariates was used to determine the association between the study groups and the risk to develop T2DM. For all analyses, *p* ≤ 0.05 was considered to be statistically significant. All analyses were performed using the statistical package SPSS 22.0 for Windows (SPSS, Chicago, IL, USA).

## 3. Results

Table 1 represents the anthropometric, biochemical, and metabolic characteristics of subjects stratified according to ADA criteria for the classification of glucose tolerance [30]. No significant differences between the three study groups were observed with respect to gender (*p* = 0.62). As reported in Table 1, the subjects in the NGT group were younger (*p* < 0.0001 versus IFG/IGT and T2DM) and showed a significantly better profile for metabolic parameters, such as FPG (*p* < 0.0001 versus IFG/IGT and T2DM), 2-h post load plasma glucose (*p* < 0.0001 versus IFG/IGT and T2DM), insulin levels (*p* < 0.001 versus IFG/IGT and *p* < 0.0001 versus T2DM), HbA1c% (*p* < 0.001 versus IFG/IGT and *p* < 0.0001 versus T2DM), HOMA-IR and Matsuda Index (each with *p* < 0.0001 versus IFG/IGT and T2DM), after adjusting for age, sex, and BMI. Circulating Mg^2+^ levels were significantly lower in the T2DM group (*p* < 0.01 versus NGT and *p* < 0.02 versus IFG/IGT). No significant differences were reported for the lipid profile even though triglycerides were significantly increased in IFG/IGT (*p* < 0.01) and newly diagnosed T2DM groups (*p* < 0.001). SBP appeared significantly better in the NGT group (*p* < 0.01 versus IFG/IGT and T2DM) and no difference was found for DBP values (*p* = 0.06). Finally, the InsAUC30/GluAUC30 and the Stumvoll first-phase indexes of insulin secretion, estimated from the OGTT, showed differences among the three groups of individuals. In detail, the NGT group had a better b-cell function with respect to IFG/IGT and T2D when the Stumvoll first-phase index (*p* < 0.05 and *p* < 0.01, respectively) and InsAUC30/GluAUC30 (*p* < 0.01 and *p* < 0.01, respectively) were analyzed (Table 1).

In the univariate analyses adjusted for the same covariates (Table 2), circulating Mg^2+^ levels correlated positively with Tot-Col (r = 0.151, *p* < 0.001), HDL-Col (r = 0.103, *p* < 0.01), and LDL-Col (r = 0.164, *p* < 0.0001) while, more interestingly, we report a negative correlation with HbA1c (r = −0.101, *p* < 0.001), FPG (r = −0.112, p < 0.01), 2-h PG (r = −0.122, *p* < 0.01), and BMI (r = −0.105, *p* < 0.01). 

To estimate the independent contribution of Mg^2+^, on the indices of insulin secretion (Stumvoll first- and InsAUC30/GluAUC30), we conducted a multivariate regression analysis in a model also including age, sex, BMI, systolic and diastolic blood pressure, triglycerides, total and HDL cholesterol (Table 3). The variables that remained significantly associated with InsAUC30/GluAUC30 were age (β = −0.333; *p* < 0.0001), BMI (β = 0.239; *p* < 0.0001), and Mg^2+^ (β = 0.08; *p* < 0.04). Similarly, when we use the Stumvoll first-phase as dependent variable in a multivariate regression analysis we found significantly associated the age (β = −0.309; *p* < 0.0001), BMI (β = 0.290; *p* < 0.0001), and Mg^2+^ (β = 0.108; *p* < 0.01). No differences were reported when dyslipidemic therapy, presence of hypertension, and diuretics use were included in the analysis. 

To estimate the independent contribution of Mg^2+^ to the risk of T2DM, we performed a logistic regression analysis, which is reported in Table 4. In the first model (Model 1) only age, gender, BMI, and Mg^2+^ were included as confounding variables, resulting in a nominally significant association with reduced odds of developing diabetes (OR = 0.844 CI 95% 0.727–0.980, *p* < 0.03). In Model 2, which additionally included the presence of family history of diabetes, hypertension, dyslipidemic therapy, or diuretics, Mg^2+^ was significantly associated with the reduced onset of T2DM (OR = 0.836 CI 95% 0.719–0.972, *p* < 0.02). Since increased renal Mg^2+^ excretion may result from uncontrolled glycemic status, therefore causing lower circulating Mg^2+^ levels, the subsequent analysis was performed in the presence of e-GFR. Both the association and its direction remained consistent with what was obtained in Model 2 (OR = 0.765 CI 95% 0.629–0.932, *p* < 0.01) (Model 3).

Among the whole study population, 365 non-diabetic subjects (almost 62% of the baseline population) were longitudinally studied over a FU period (mean FU, 5.6 ± 0.8 years) to evaluate the incidence of T2DM (Table 5). To estimate the hazard ratio (HR) of Mg2+ in the onset of T2DM, a Cox regression analysis including age, sex, BMI, glucose tolerance status, smoking habits, presence of hypertension, dyslipidemia, and relative treatments (i.e., diuretics and statins) has been performed. As indicated in Table 5, we observed an HR = 0.764 with 95% CI = 0.636–0.918 (*p* < 0.01) (Model 1), explaining the detrimental effect of low levels of circulating Mg^2+^. After adding to the Model 1 therapies for hypertension, dyslipidemia, or diuretics, tolerance status at basal, the presence of a family history of diabetes and e-GFR, the analysis remained significant (HR = 0.790 with 95% CI = 0.645–0.967; *p* = 0.022).

## 4. Discussion

Mg^2+^ can be found in many foods such as grains, nuts, and green leafy vegetables, and it plays a key role in many fundamental biological processes, as well as in glucose metabolism [7]. Over the past two decades clinical evidence have been reported, showing that depletion of serum Mg^2+^ increases exponentially with the duration of T2DM [35]. Diabetes is associated with low Mg^2+^ [24], and hypomagnesemia is associated with insulin resistance, inflammation, and increased risk for cardiovascular disease [36].

At the molecular level, it has been suggested that hypomagnesemia may induce altered cellular glucose transport, defective tyrosine-kinase activity, and insulin receptor autophosphorylation, post-receptor impairment in insulin action by influencing intracellular signaling cascade and processing, reduced pancreatic insulin secretion, and worsening of insulin resistance in diabetics [37,38,39,40]. Above all, defective insulin receptor phosphorylation is regarded as the main mechanism by which hypomagnesemia contributes to insulin resistance in T2DM patients [24], and some epidemiological studies have suggested that adequate Mg^2+^ intake may reduce T2DM incidence [41].

On the other hand, insulin is known to stimulate the transport of Mg^2+^ from the extracellular to the intracellular compartment. Therefore, T2DM could contribute to the reduction of serum Mg^2+^ levels, and this could, in turn, worsen glycemic control of diabetes. The chronicity of this vicious cycle has been suggested to accelerate the impairment of metabolic control and to predispose to chronic complications of T2DM [36,38,42,43]. 

This project supplies evidences of this complex phenomenon. In our analysis we acquired a significant negative correlation between Mg^2+^ levels, fasting glucose, and 2h-post load glucose in subjects who underwent an OGTT. Moreover, Mg^2+^ levels correlated negatively with fasting insulin levels, and positively with the lipid profile. As for the detrimental effect of lower circulating Mg^2+^ levels, a Cox proportional hazard regression analysis revealed a significant reduction of T2DM risk of about 20% for each 1 mg/dL increase of circulating Mg^2+^.

The multivariate regression analysis showed a strong and significant association between serum Mg^2+^ levels with InsAUC30/GluAUC30 and the first phase of insulin secretion for the overall group. It has been proposed that the decrease in insulin sensitivity precedes the impairment of b-cell function, and that the impairment of b-cells to compensate for insulin resistance is a late phenomenon [44].

The present study has several strengths including the large sample size and the homogeneity of the patient group, the detailed characterization of the study population, the centralization of biochemical analyses, and the exclusion of differences in dietary habits and nutritional conditions. Unfortunately, it was not possible to estimate the daily food and water intake in terms of Mg^2+^, during the follow-up period, but overall, during the follow-up visit, the subjects referred no variations in their dietary habits.

Other solid aspects are the prolonged time of follow-up and the exclusion of residual confounding, although outcomes were adjusted for a wide range of potential confounders. At the same time, the geographical and ethnical restriction of the population should be considered as a limit to the generalizability of the data: All participants to the study were Caucasians from Southern Italy, and whether the present findings can also be extended to non-Caucasian ethnic groups will require extensive evaluation in the future. 

One limitation of our study is the impossibility to address the question of at what point in the natural history of type 2 diabetes do Mg^2+^ levels start to decrease. The best way to address this issue would probably be to compare repeated measurements of Mg^2+^ across a time period. In future research, this approach would allow grouping of similar trajectories within homogenous subgroups, while limiting the within-person variability. In addition to this, we acknowledge that serum Mg^2+^ is not a robust predictor of type 2 diabetes risk, and that it is possible that the small differences in serum Mg^2+^ levels observed in our cohort might be amplified in other districts (e.g., intracellular and interstitial compartments), but we have no information about this issue.

The present results are consistent with the theory that Mg^2+^ supplementation could ameliorate insulin sensitivity reducing the risk to develop T2DM. Oral Mg^2+^ supplements appear to be useful in persons with T2DM to restore Mg^2+^ deficiencies, to improve insulin resistance, oxidative stress, and systemic inflammation [45]. This finding suggests the need for screening serum magnesium levels in the subjects in the high-risk groups for developing glucose metabolic disorders. However, the picture is still far from complete, and requiring confirmation by further studies to fully understand the complex and dynamic effect of Mg^2+^ in T2DM and to elucidate the role of the nutrient as a protector factor against metabolic disorders.

## Figures and Tables

**Table 1:** Anthropometric and metabolic characteristics of the study subjects stratified according to American Diabetes Association (ADA) criteria for the classification of glucose tolerance.

Variables	Whole Study Group	NGT (1)	IFG/IGT (2)	T2D (3)	*p*	*p*	*p*	*p*
						(1 vs. 2)	(1 vs. 3)	(2 vs. 3)
Gender (M/F)	288/301	146/207	108/77	34/17	0.62	—	—	—
Age (years)	47 (±13)	44 (±14)	51 (±11)	55 (±12)	<0.0001 *	<0.0001	<0.0001	<0.04
BMI (Kg/m^2^)	30.7 (±7.1)	30.1 (±7.2)	31.5 (±6.9)	31.8 (±6.9)	<0.001 **	<0.001	<0.01	0.47
SBP (mmHg)	124.4 (±16.1)	120.9 (±15.1)	128.7 (±15.8)	132.4 (±17.2)	<0.01	<0.01	<0.01	0.41
DBP (mmHg)	77.5 (±10.3)	76.4 (±10.4)	78.4 (±9.6)	81.5 (±11.3)	0.06	0.28	<0.03	0.11
Tot-COL (mg/dL)	195.2 (±40.6)	193.0 (±39.6)	199.7 (±37.0)	193.9 (±56.1)	0.24	0.10	0.94	0.36
HDL-Col (mg/dL)	50.8 (±14.1)	53.1 (±13.9)	48.0 (±13.9)	45.6 (±13.2)	<0.01	<0.01	<0.02	0.41
LDL-Col (mg/dL)	123.9 (±35.4)	121.7 (±35.2)	128.3 (±31.2)	123.4 (±48.2)	0.33	0.18	0.84	0.31
Triglycerides (mg/dL)	127.6 (±77.0)	113.1 (±68.4)	144.3 (±80.7)	169.4 (±92.3)	<0.001	<0.01	<0.001	0.07
Mg^2+^ (mg/dL)	1.99 (±0.18)	2.01 (±0.17)	1.99 (±0.18)	1.92 (±0.21)	<0.02	0.66	<0.01	<0.01
HbA1c (%)	5.60 (±0.67)	5.38 (±0.32)	5.65 (±0.37)	6.94 (±1.39)	<0.0001	<0.001	<0.0001	<0.0001
HOMA-IR	3.53 (±3.02)	2.89 (±2.07)	3.89 (±2.09)	6.69 (±7.09)	<0.0001	<0.001	<0.0001	<0.0001
e-GFR, mL/min/1.73/m^2^	133.8 (±38.6)	142.6 (±39.3)	122.2 (±34.2)	117.5 (±33.5)	0.84	0.71	0.75	0.59
Fasting glucose (mg/dL)	94.4 (±13.2)	88.8 (±7.5)	100.1 (±10.9)	112.1 (±23.7)	<0.0001	<0.0001	<0.0001	<0.0001
2-h glucose (mg/dL)	127.5 (±38.4)	107.3 (±22.3)	147.3 (±24.6)	209.1 (±45.4)	<0.0001	<0.0001	<0.0001	<0.0001
FP insulin (mU/ml)	14.9 (±12.1)	13.2 (±9.4)	15.6 (±8.1)	24.1 (±28.0)	<0.0001	<0.003	<0.0001	<0.0001
Stumvoll 1^st^-phase Index	1722 (±1002.5)	1855 (±1034.1)	1547 (±939.5)	1361 (±782.1)	<0.02	<0.05	<0.01	0.11
InsAUC30/GluAUC30	8.3 (±5.7)	9.1 (±6.1)	7.1 (±4.7)	6.7 (±5.3)	<0.01	<0.01	<0.01	0.26
Matsuda Index	66.9 (±44.6)	79.9 (±47.5)	50.2 (±31.5)	31.3 (±19.1)	<0.0001	<0.0001	<0.0001	<0.1
Hypolipidemic Teraphy %	11.9	5.9	11.9	33.3	<0.0001	<0.0001	<0.0001	<0.01
Hypertension %	52.1	41.6	65.4	76.5	<0.0001	<0.0001	<0.0001	0.13
Diuretics %	11.9	4.2	14.6	13.7	<0.0001	<0.0001	<0.01	0.87
Family history of diabetes %	53.3	51.3	52.0	71.7	<0.03	0.92	<0.01	<0.02

Data are means ± SD. Comparisons among the three groups were performed using a general linear model with post hoc Bonferroni correction for multiple comparisons. *p* values refer to results after analyses with adjustment for age, gender, and BMI. *p values refer to results after analyses with adjustment for gender and BMI. **p values refer to results after analyses with adjustment for age and gender. Categorical variables were compared by *X*^2^ test. IGT, impaired glucose tolerance; IFG, impaired fasting glucose; SBP, systolic blood pressure; DBP, diastolic blood pressure.

**Table 2:** Univariate correlations between Mg^2^+ levels and anthropometric and metabolic variables.

Variables	Pearson’s Correlation Coefficient (*r*)	*p*
Age (years)	−0.066	0.107 *
BMI (Kg/m^2^)	−0.087	0.03 **
SBP (mmHg)	0.045	0.275
DBP (mmHg)	0.048	0.248
Tot-Col (mg/dL)	0.154	<0.001
HDL-Col (mg/dL)	0.113	<0.01
LDL-Col (mg/dL)	0.170	<0.001
Triglycerides (mg/dL)	0.01	0.981
HbA1c (%)	−0.099	0.018
Fasting glucose (mg/dL)	−0.119	<0.01
2-h glucose (mg/dL)	−0.116	<0.01
FP insulin (mU/ml)	−0.073	0.08
e-GFR, mL/min/1.73/m^2^	−0.036	0.41

Adjusted for age, gender, and BMI *Adjusted for gender and BMI; **Adjusted for age and gender. BMI = body mass index; SBP = systolic blood pressure; DBP = diastolic blood pressure; HDL = high density lipoprotein; LDL = low density lipoprotein.

**Table 3:** Multiple regression analysis with indices of insulin secretion as dependent variables.

Indices of Insulin Secretion	Independent Contributors	Standardized Coefficient β	*p*
InsAUC30/GluAUC30 *	Age	−0.333	<0.0001
	BMI	0.239	<0.0001
	Mg^2+^	0.09	<0.04
Stumvoll 1st-phase Index *	Age	−0.309	<0.0001
	BMI	0.290	<0.0001
	Mg^2+^	0.108	<0.01

* Model includes age, sex, BMI, systolic and diastolic blood pressure, triglycerides, total and HDL cholesterol.

**Table 4:** Odds ratio (95% CI) by multiple logistic regression analysis for the risk to develop T2DM.

Study Group	OR	95% CI	*p*
Model 1	0.844	0.727–0.980	<0.03
Model 2	0.836	0.719–0.972	<0.02
Model 3	0.765	0.629–0.932	<0.01

Model 1: Adjusted for age, gender, BMI, and Mg2+; Model 2: Model 1 + family history of diabetes + therapies; Model 3: Model 2 + e-GFR.

**Table 5:** Hazard ratio (HR) (95% CI) by multiple logistic regression analysis for the risk to develop T2DM.

Study Group	HR	95% CI	*p*
Model 1	0.764	0.636–0.918	<0.01
Model 2	0.790	0.645–0.967	0.022

Model 1: Adjusted for age, gender, BMI, and Mg^2+^; Model 2: Model 1 + therapies for hypertension, dyslipidemia, or diuretics + tolerance status+ family history of diabetes + e-GFR.
